# The interleukin-27 -964A>G polymorphism enhances sepsis-induced inflammatory responses and confers susceptibility to the development of sepsis

**DOI:** 10.1186/s13054-018-2180-0

**Published:** 2018-09-30

**Authors:** Junbing He, Quanfu Zhang, Wenying Zhang, Feng Chen, Tian Zhao, Yao Lin, Jia Li, Yansong Liu, Yuchun Liu, Yiming Shao

**Affiliations:** 1 The Intensive Care Unit, Jieyang Affiliated Hospital, SunYat-sen University, Tianfu Road 107, Rongcheng District, Jieyang City, 522000 Guangdong Province People’s Republic of China; 2 The Intensive Care Unit, Affiliated Hospital of Guangdong Medical University, Renmin Street South 57, Xiashan District, Zhanjiang City, 524001 Guangdong Province People’s Republic of China; 3 The Department of Gynecology and Obstetrics, Shenzhen Maternity and Child Healthcare Hospital Affiliated to Southern Medical University, Shenzhen, Guangdong People’s Republic of China; 4 The Intensive Care Unit, the Central Hospital of Wuhan, Tongji Medical College, Huazhong University of Science and Technology, Hubei, People’s Republic of China; 5 The Intensive Care Unit, the Second Affiliated Hospital of Harbin Medical University, Harbin, People’s Republic of China

**Keywords:** IL-27, Polymorphism, Sepsis, Inflammation, TNF-α, IL-1β, IL-6

## Abstract

**Background:**

Previous studies have identified critical roles of IL-27 in the pathological mechanisms of sepsis, and blockade of IL-27 may be a promising alternative therapy for sepsis. The purpose of this study was to evaluate the clinical relevance of *IL-27* genetic polymorphisms in sepsis and to further characterize their effect on *IL-27* expression and inflammatory processes following sepsis.

**Methods:**

A total of 885 septic patients and 1101 healthy controls were enrolled and genotyped for *IL-27* genetic variants (rs153109/−964A > G and rs17855750/2905 T > G). Quantitative real-time PCR and enzyme-linked immunosorbent assays were performed to detect *IL-27* expression and cytokine production. The effect of the rs153109 polymorphism on *IL-27* promoter activity was evaluated using a luciferase reporter assay, and THP-1 cell apoptosis was calculated using an annexin V apoptosis assay.

**Results:**

No significant differences in the genotype/allele frequencies were observed between patients with sepsis and healthy controls, suggesting that these two *IL-27* polymorphisms may not influence susceptibility to sepsis. The -964AA genotype was overrepresented in patients with severe sepsis/septic shock relative to patients with the sepsis subtype, and the A allele was significantly associated with 28-mortality in sepsis. Patients carrying the -964AA genotype exhibited significantly higher expression levels of *IL-27* than the GA/GG genotype carriers. The results of an in vitro (lipopolysaccharide (LPS))-stimulated experiment showed that this sepsis-associated high-risk AA genotype significantly increased IL-27 levels and enhanced TNF-α and IL-1β production in the peripheral blood mononuclear cells (PBMCs) upon exposure to LPS in vitro. Furthermore, luciferase reporter assays indicated that the high-risk -964A allele resulted in increased promoter activities compared to the non-risk allele in THP-1 and 293 T cells. Additionally, IL-27 treatment significantly enhanced TNF-α and IL-6 secretion and apoptosis of THP-1 cells upon LPS stimulation.

**Conclusions:**

These results provided evidence that the *IL-27* -964A > G polymorphism functionally enhanced *IL-27* expression and promoted sepsis-induced inflammatory responses, which ultimately resulted in promoting the progression of sepsis and poor prognosis.

**Electronic supplementary material:**

The online version of this article (10.1186/s13054-018-2180-0) contains supplementary material, which is available to authorized users.

## Background

Sepsis is a multisystem disease that usually results from infection and is mediated by severe systemic inflammatory responses. This disease can lead to hemodynamic alterations, multiple organ dysfunction and septic shock [1, 2]. Despite extensive progress in the management of sepsis and critical care techniques, sepsis-associated mortality remains as high as 30–50% [3]. Growing evidence indicates that genetic polymorphisms in genes encoding inflammation-related cytokines, including IL-6, IL-10 and TNF-α, play significant roles in the pathogenesis of sepsis and even contribute to sepsis susceptibility and progression [4–6]. Identifying the single nucleotide polymorphisms (SNPs) in these genes and associated differences in detrimental systemic inflammatory responses may facilitate the identification of and progress in genetic diagnostic and new therapeutic targets of sepsis that will improve the prognosis of critically ill patients.

Interleukin-27 (IL-27), a recently identified heterodimeric cytokine belonging to the IL-12 family, is composed of two subunits of IL-27p28 and Epstein-Barr virus (EBV)-induced gene 3 (*EBI3*) and is secreted primarily by stimulated antigen-presenting cells, such as macrophages and dendritic cells [7, 8]. IL-27 has emerged as a pro-inflammatory factor that signals via binding to IL-27R, which consists of IL-27Ra (WSX-1/TCCR) and glycoprotein 130 subunits, and mediates various inflammation-promoting biological activities involved in the pathogenesis of many inflammation-related diseases, including sepsis [9–11]. Recent studies have indicated that IL-27 expression is significantly increased in patients with sepsis and in septic mice, and is strongly correlated with disease severity and mortality following sepsis [12, 13]. *IL-27R* knock-out in mice shows increased resistance against secondary bacterial pneumonia in sepsis and protection against sepsis-induced lethality, whereas treatment with recombinant IL-27 confers susceptibility to *Pseudomonas aeruginosa* infection and enhanced lipopolysaccharide (LPS)-induced pro-inflammatory cytokine production [14–16]. Other studies have demonstrated that the neutralization of IL-27 decreases the inflammatory response and affords mice a survival benefit following sepsis [17, 18]. These findings indicate the critical role of IL-27 in the pathological mechanisms of sepsis, and blockade of IL-27 may be a promising alternative therapy for sepsis.

The human *IL-27* gene is located on chromosome 16p11 and contains five exons. Recently, two genetic polymorphisms of *IL-27*, namely, rs153109 and rs17855750, were reported to be associated with a variety of diseases [19, 20]. The rs153109 (− 964 A > G) polymorphism located 964 bp upstream of the transcription start site of the *IL-27* gene has been demonstrated to be a potentially functional polymorphism that may alter transcription and expression of *IL-27*, leading to a genetic predisposition to many inflammatory diseases, such as ulcerative colitis, chronic obstructive pulmonary disease (COPD) and rheumatoid arthritis [21–23]. Another polymorphism in our study, rs178855750 (2905 T > G) is located in exon 2 of the *IL-27* gene and has also been implicated in allergic rhinitis and cervical cancer [24, 25]. Nonetheless, there are currently no studies exploring the genetic association of these *IL-27* SNPs with sepsis, and the related mechanisms by which these variants influence systemic inflammatory responses upon sepsis remain to be elucidated.

In this study, 885 patients with sepsis and 1101 healthy subjects were examined to evaluate the clinical relevance of two *IL-27* genetic SNPs, rs153109 (− 964 A > G) and rs17855750 (2905 T > G), in the susceptibility and progression of sepsis in a Han Chinese population. Furthermore, functional in vitro assays were performed to characterize the effect of these *IL-27* variants on its gene expression and inflammatory processes following sepsis. The mechanistic characterization of this sepsis-associated functional polymorphism might provide new opportunities for the development of targeted treatments for sepsis.

## Methods

### Subject enrollment

This study was approved by the Ethical Committee of the Affiliated Hospital of Guangdong Medical University (Zhanjiang, China), the Central Hospital of Wuhan (Hubei, China) and the Second Affiliated Hospital of Harbin Medical University (Harbin, China), and performed according to the Declaration of Helsinki. Written, signed, informed consent was provided by all enrolled subjects or their families prior to enrollment. The diagnosis of sepsis, severe sepsis, or septic shock was established according to the International Sepsis Definitions Conference [26, 27]. Patients with sepsis who suffered from diabetes mellitus, malignant tumors, human immunodeficiency virus, or autoimmune diseases or who were receiving immunosuppressive, steroid, or radiation therapy were excluded from this study. The healthy controls were free from any recent acute illness, any chronic illness such as autoimmune diseases, hypertension, diabetes mellitus, COPD, cancer or major cardiac, renal, hepatic, or endocrinological disorders, and any history of sepsis according to our previous study [28]. The health conditions were determined by examining the medical examination reports; and by questioning the participators or their next of kin. Initially, 605 patients who were suffering from sepsis in the intensive care unit (ICU) within 24 h of admission, and 769 healthy controls matched for age and gender, from the Health Examination Center at the Affiliated Hospital of Guangdong Medical University were enrolled into cohort I, between December 2012 and February 2018. In order to verify the results of genotype analysis in cohort I, another cohort of 249 subjects (110 patients with sepsis and 139 matched healthy controls) from the Central Hospital of Wuhan and 363 subjects (170 patients with sepsis and 193 matched healthy controls) from the Fourth Affiliated Hospital of Harbin Medical University were enrolled as cohort II, between April 2017 and March 2018. A total of 885 patients with sepsis and 1101 controls in the cohorts I + II were older than 18 years of age and were from the Chinese Han population. The age, gender, source of infection, dysfunctional organs, blood microbiological cultures, and Acute Physiology and Chronic Health Evaluation (APACHE) II score of each patient were documented, and their blood samples were collected in the 12-h period after the diagnosis of sepsis.

### DNA extraction and genotyping for the *IL-27* SNPs

Genomic DNA from the peripheral blood was extracted via using a TIANamp DNA extraction kit (Tiangen Biotech, Beijing, China) and stored at − 80 °C until used. The ABI PRISM SNaPshot method (Applied Biosystems, Carlsbad, CA, USA) was performed to genotype the two *IL-27* SNPs rs153109 and rs17855750. The primers used for PCR amplification of two target fragments (227 and 232 bp, respectively) were as follows: rs153109F, 5′ CCCGGCTGTGTCTGTGTTTAGG 3′; rs153109R, 5′ GGTTGAGCCCTGATCCTGACCT 3′; rs17855750F, 5′ CTGGGGGGCAAGGTCTGTTAGT 3′; rs17855750R, 5′ GCTCCTGGTTCAAGCTGGTGTC 3′. Also, 10% of samples were chosen at random for re-genotyping as an independent validation group. The experimental results were analyzed using GeneMapper 4.1 (Applied Biosystems, Carlsbad, CA, USA).

### Plasma collection, mononuclear cell isolation and LPS stimulation

The peripheral blood mononuclear cells (PBMCs) were isolated from the patients with sepsis and healthy individuals by density gradient centrifugation with Lymphoprep™ (Axis-Shield PoCAS, Oslo, Norway). Plasma was also extracted from the blood samples by centrifugation at low speed and was stored at − 80 °C until used for the cytokine measurements. The PBMCs from the 55 healthy individuals were randomly selected for LPS stimulation experiments in vitro. Briefly, 1 × 10^5^ cells were cultured in RPMI 1640 (Thermo Fisher Scientific, Waltham, MA, USA) contained with FBS (Thermo Fisher Scientific) in 24-well plates. After stimulation with 500 ng/mL of LPS for 8 h, the cells and supernatants were collected for real-time (RT)-PCR analysis and cytokine measurements, respectively. The control cells were treated with the PBS vehicle.

### RNA extraction and quantitative RT-PCR

For RT-PCR analysis of *IL-27* messenger RNA (mRNA) expression, TRIzol reagent (Sangon Biotech, Shanghai, China) was used to isolate total RNA from PBMCs, which were derived from 160 randomly selected subjects (80 patients with sepsis and 80 healthy controls) or from the LPS stimulation experiments in vitro. Then a First Strand complementary DNA (cDNA) Synthesis Kit (Thermo Fisher Scientific) was used to convert the RNA into cDNA following the instructions of the manufacturers. Quantitative RT-PCR was conducted to detect the expression levels of *IL-27* using the SYBR Green RT-PCR Kit (Takara). The human ACTB (*β-actin*) gene was used as a control in the quantitative RT-PCR analysis. The primer sequences used in this study were designed by Sangon Biotech with the Primer Premier 5.0 program (Applied Biosystems) as follows: *IL-27*: 5’ CGGAGGGAGTTCA CAGTCAG 3′ and 5′ CAGGTGAGATTCCGCAAAGC 3′; *β-actin*: 5′ TCCCTGGAGAAGAGCTACGA 3′ and 5′ AGCACTGTGTTGGCGTACAG 3′. Amplification was performed in a LightCycler480 sequence detector system (Roche Applied Science, Laval, QC, Canada) with 40 cycles of 5 s at 95 °C, 20s at 58 °C, and 1 min at 72 °C. The *IL-27* mRNA expression was calculated using the 2^-△△CT^ method with values normalized to *β-actin* expression.

### Plasmid construction and luciferase reporter assay

According to our Initial results, a 2145-bp promoter sequence (− 2000 to + 145) carrying rs153109 (A or G allele at position − 964) of *IL-27* gene (*Homo sapiens* chromosome 16: 28,499,362-28,506,834) was cloned into pGL3 luciferase reporter vectors (Promega, Madison, WI, USA). The promoter gene fragment of *IL-27* was created by PCR amplification using the *IL-27* primer sequences as follows: 5′-TCCACGCGTGAGACAGTGTCTTGCTCTGTTG-3′ (forward) and 5′-GCCAAGCTTGCCAGCCAAGGTCGCCTGC-3′ (reverse). The THP-1 and 293 T cells and human umbilical vein endothelial cells (HUVEC) obtained from Shanghai Institute of Cell Biology (Shanghai, China) were transfected with the pGL3-basic original plasmid or the constructed vectors using Lipofectamine 2000 (Invitrogen, USA) following the instructions of the manufacturer. The assay was conducted follow the protocol of the dual-luciferase assay kit (Promega, USA), and measured in the Mithras LB940 Multilabel Reader (Berthold Technologies, Bad Wildbad, Germany).

### IL-27 and LPS treatment experiments

THP-1 cells were cultured in RPMI 1640 medium (Thermo Fisher Scientific) contained with 10% FBS (Thermo Fisher Scientific) at 37 °C. IL-27 was purchased from MedChem Express (Monmouth Junction, NJ, USA). Cells were treated with IL-27 (100 ng/mL), LPS (500 ng/mL), or LPS plus IL-27 for 8 and 16 h. At each time point, the THP-1 cells and supernatants were collected for annexin V apoptosis assay and cytokine measurements, respectively.

### Cytokine measurements

The plasma from 160 randomly selected subjects (80 patients with sepsis and 80 controls), and culture supernatants from the in vitro experiment were used to detect expression of the cytokines IL-27, IL-1β, IL-6 and TNF-α, following the manufacturer’s instructions for each specific enzyme-linked immunosorbent assay (ELISA) kit (TianGen Biotech). Absorbance was measured at 450 nm with a microplate reader.

### Annexin V apoptosis assay

The apoptosis of THP-1 cells was detected using the ANXA5/AnnexinV-FITC Apoptosis Detection Kit (Beyotime institute of biotechnology, Shanghai, China) following the manufacturer’s instructions. Briefly, cells were collected and washed twice with PBS and resuspended in 195 μL binding buffer. Then 10 μL propidium iodide (PI) and 5 μL ANXA5-FITC stock solution were added for 15 min of incubation at 37 °C, protected from light. Then the apoptosis of cells was immediately analyzed by FACS.

### Statistical analyses

The association between *IL-27* polymorphisms and sepsis was analyzed using the chi-squared test or Fisher’s exact test. The false discovery rate was analyzed using the Benjamini-Hochberg correction for multiple testing. Power analysis calculated using the QUANTO 1.2 program (University of Southern California, LA, USA) showed 100% and 79.3% power with a significance level of 0.05 at an odds ratio of 1.5 for rs153109 and rs17855750 on the basis of the sample size, respectively. The Kaplan-Meier method was used to plot 28-day survival curves, using the different genotypes of the *IL-27* polymorphisms, and these were compared using the log-rank test. *IL-27* mRNA expression and cytokine levels in the independent groups were compared using the non-parametric Mann-Whitney U test. All data are presented as the mean ± standard error of the mean (SEM). Statistical analysis was conducted using SPSS version 19.0 (IBM, NY, USA) and GraphPad Prism 4.0 (GraphPad Software Inc., San Diego, CA, USA). A *P* value less than 0.05 was considered statistically significant.

## Results

### The characteristics of the study population

The Consolidated Standards of Reporting Trials (CONSORT) flowchart of this study is shown in Additional file 1. The demographic and clinical characteristics of the subjects studied (885 patients with sepsis and 1101 healthy controls) are summarized in Table 1. A total of 1986 subjects were successfully genotyped for rs153109 and enrolled in this study. Only 403 patients with sepsis and 400 controls in the initial cohort I were genotyped for rs17855750. No significant differences in age or gender distribution were detected between patients with sepsis and healthy controls in cohorts I, II and I + II (Table 1, all *P* > 0.05). The primary source of infection in these two study cohorts was lung infection: 18.3% and 47.1% of cohorts I + II had Gram-positive and Gram-negative infection, respectively. Patients with polymicrobial infections made up 21.9% of these two study cohorts. The primary pathogenic bacteria identified in this study were *Acinetobacter baumannii* (24.9%), *P*. *aeruginosa* (12.3%), *Escherichia coli* (10.6%), *Klebsiella pneumoniae* (7.5%), and *Staphylococcus aureus* (6.8%). The main comorbidities in patients with sepsis included severe pneumonia (22.4%), hypertension (19.8%), cerebrovascular disease (17.3%), trauma (13.7%), renal disease (10.3%), and COPD (10.1%), as shown in Additional file 2. The 885 patients with sepsis comprised 18.6% with sepsis subtype, 41.2% with severe sepsis, and 40.2% with septic shock. The 28-day mortality rate in the whole study cohort was 24.4%.

**Table 1:** Clinical characteristics of patients with sepsis and healthy controls

Variable	Sepsis (*n* = 885) Number (percent)	Control (*n* = 1101) Number (percent)	*P* value
Demographics			
Age, years, mean ± SEM	60.05 ± 0.58	59.02 ± 0.52	0.189
Male/female, *n*	584/301	688/413	0.106
Sepsis status, *n* (%)			
Sepsis subtype	165 (18.6)	N.A	
Severe sepsis	364 (41.2)	N.A	
Septic shock	356 (40.2)	N.A	
Source of infection, *n* (%)			
Respiratory tract infection	629 (71.1)	N.A	
Primary bloodstream infection	126 (14.2)	N.A	
Abdominal infection	193 (21.8)	N.A	
Urinary tract infection	45 (5.1)	N.A	
Catheter-associated infection	51 (5.8)	N.A	
Brain	37 (4.2)	N.A	
Others	60 (6.8)	N.A	
Infection type, *n* (%)			
Gram-positive	162 (18.3)	N.A	
Gram-negative	417 (47.1)	N.A	
Mixed Gram-negative and Gram-positive	163 (18.4)	N.A	
Fungus	136 (15.3)	N.A	
Polymicrobial	194 (21.9)	N.A	
Negative blood culture	59 (6.7)	N.A	
Pathogenic bacteria, *n* (%)			
*Acinetobacter baumannii*	220 (24.9)	N.A	
*Monilia albican*	47 (5.3)	N.A	
Yeast sample sporphyte	34 (3.8)	N.A	
Aspergillus	30 (3.4)	N.A	
*Klebsiella pneumoniae*	66 (7.5)	N.A	
*Pseudomonas aeruginosa*	109 (12.3)	N.A	
*Staphylococcus aureus*	60 (6.8)	N.A	
*Escherichia coli*	94 (10.6)	N.A	
Others	169 (19.1)	N.A	
APACHE II score	25.6 ± 5.7	N.A	
Mortality, 28 days, *n* (%)	216 (24.4)	N.A	

Continuous data are expressed as the mean ± SEM

*N.A* not applicable*, APACHE II* Acute Physiology and Chronic Health Evaluation II

### The association between *IL-27* SNPs and sepsis susceptibility and progression

**Fig. 1:**
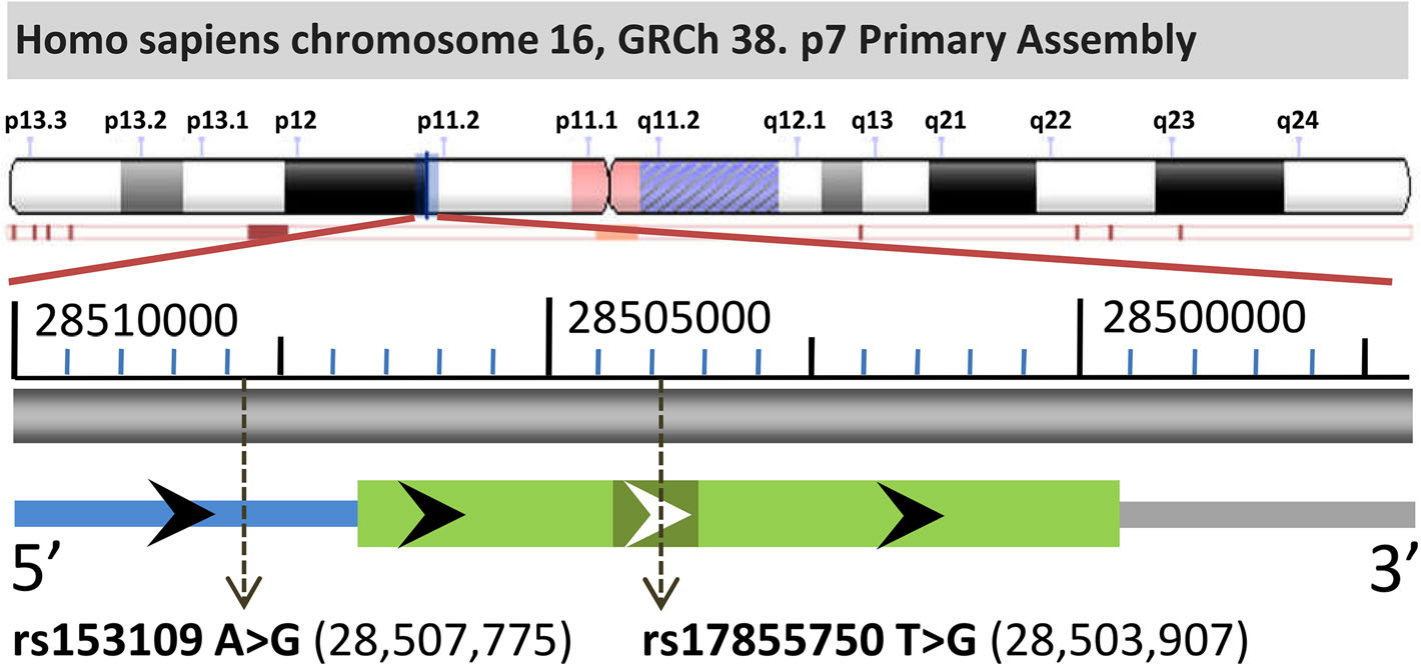
The *IL-27* polymorphisms and their locations in the *IL-27* gene. According to the GRCh38.p7 primary assembly, the human *IL-27* is encoded in *Homo sapiens* chromosome 16 (28,499,362-28,506,834). Its exon2 is shown as a dark green bar. The blue bar represents the 5’ UTR of the *IL-27* gene. rs153109 (−964A > G) and rs17855750 (2905 T > G) are located upstream of the transcription start site (−964 bp) and into the exon2 of the *IL-27* gene, respectively

Two *IL-27* SNPs, rs153109 in the promoter region and rs17855750 in exon 2 of the gene are presented in Fig. 1. The genotype distributions of these two *IL-27* SNPs in both the patients and controls were consistent with Hardy-Weinberg equilibrium (all *P* > 0.05, see Additional file 3). No significant differences in the genotype/allele frequencies of rs153109 and rs17855750 were found between patients with sepsis and healthy controls in study cohorts I, II and I + II, suggesting that these two *IL-27* SNPs might not influence susceptibility to sepsis (Tables 2 and 3, all *P* > 0.05). We further divided the patients with sepsis into subgroups of sepsis subtype, severe sepsis or septic shock, based on the severity of sepsis, to assess the effect of *IL-27* SNPs on the development of sepsis. As shown in Table 4, the frequencies of the AA genotypes and A allele at the *IL-27* SNP rs153109 A > G were significantly greater in the severe sepsis subgroup (*P* = 0.002 for genotype and allele) and septic shock subgroup (*P* = 0.0002 for genotype and *P* = 0.0003 for allele) compared to the sepsis subtype subgroup, which indicated a significant role of A allele in promoting the progression of sepsis from sepsis subtype to severe sepsis/septic shock. Nevertheless, no significant differences were observed among the sepsis subgroups in the *IL-27* SNP at rs17855750 G > T in this study (Table 4, all *P* > 0.05).

**Table 2:** Genotype and allele frequencies distribution of the *IL-27* rs153109 polymorphism in the cases and controls of cohorts I and II

	Cohort I + II				Cohort I				Cohort II		
Cases *n* (%)	Control *n* (%)	*P*	OR (95% CI)	Cases *n* (%)	Controls *n* (%)	*P* OR (95% CI)		Cases *n* (%)	Controls *n* (%)	*P* OR (95% CI)	
AA 334 (37.7)	417 (37.9)	0.885	–	233 (38.5)	276 (35.9)	0.605	–	101 (36.1)	141 (42.5)	0.183	–
AG 408 (46.1)	498 (45.2)	–	–	267 (44.1)	355 (46.2)	–	–	141 (50.3)	143 (43.1)	–	–
GG 143 (16.2)	186 (16.9)	–	–	105 (17.4)	138 (17.9)	–	–	38 (13.6)	48 (14.4)	–	–
AA/AG 742 (83.8)	915 (83.1)	0.661	1.06 (0.83–1.34)	500 (82.6)	631 (82.1)	0.776 1.04 (0.79–1.38)		242 (86.4)	284 (85.5)	0.753 1.08 (0.68–1.70)	
AG/GG 551(62.3)	684 (62.1)	0.951	1.01 (0.84–1.21)	372 (61.5)	493 (64.1)	0.318 0.89 (0.72–1.11)		179 (63.9)	191 (57.5)	0.107 1.31 (0.94–1.81)	
A 1076 (60.8)	1332 (60.5)	- 1.00 (reference)		733 (60.6)	907 (59.0)	- 1.00 (reference)		343 (61.3)	425 (64.0)	- 1.00 (reference)	
G 694 (39.2)	870 (39.5)	0.847	0.99 (0.87–1.12)	477 (39.4)	631 (41.0)	0.394 0.94 (0.80–1.09)		217 (38.7)	239 (36.0)	0.320 1.13 (0.89–1.42)	

*OR* odds ratio, *95% CI* 95% confidence interval

**Table 3:** Genotype and allele frequencies distribution of the *IL-27* rs17855750 polymorphism in the cases and controls

*IL-27*	Sepsis *n* = 403 (%)	Control *n* = 400 (%)	*P*	P*	OR (95% CI)
TT	312 (77.4)	315 (78.7)	0.719	0.719	–
TG	82 (20.4)	79 (19.8)	–	–	–
GG	9 (2.2)	6 (1.5)	–	–	–
TT/TG	394 (97.8)	394 (98.5)	0.443	0.719	0.67 (0.24-1.89)
TG/GG	91 (22.6)	85 (21.3)	0.649	0.719	1.08 (0.77-1.51)
T	706 (87.6)	709 (88.6)	–	–	1.000 (reference)
G	100 (12.4)	91 (11.4)	0.523	0.719	1.10 (0.82-1.49)

*OR* odds ratio, *95% CI* 95% confidence interval

*False discovery rate-adjusted *P* value for multiple hypotheses testing using the Benjamini-Hochberg method

**Table 4:** Genotype and allele frequencies distribution of the *IL-27* polymorphisms by different sepsis status

	Sepsis subtype *n* (%)	Severe sepsis *n* (%)	Septic shock *n* (%)	P1	P2	P1*	P2*
rs153109 (−964A > G)							
AA	42 (25.5)	142 (39.0)	150 (42.1)	0.002	0.0002	0.002	0.0003
GA/GG	123 (74.5)	222 (61.0)	206 (57.9)	–	–	–	–
A	171 (52.2)	452 (62.1)	453 (63.6)	0.002	0.0003	0.002	0.0003
G	159 (48.8)	276 (37.9)	259 (36.4)	–	–	–	–
rs17855750 (2905 T > G)							
TT	59 (73.8)	144 (79.1)	109 (77.3)	0.338	0.552	0.338	0.711
GT/GG	21 (26.2)	38 (20.9)	32 (22.7)	–	–	–	–
T	137 (85.6)	324 (89.0)	245 (86.9)	0.272	0.711	0.338	0.711
G	23 (14.4)	40 (11.0)	37 (13.1)	–	–	–	–

*P1* sepsis subtype versus severe sepsis, *P2* sepsis subtype versus septic shock

*False discovery rate-adjusted *P* value for multiple hypotheses testing using the Benjamini-Hochberg method

### The association between *IL-27* SNPs and 28-day mortality in patients with sepsis

**Fig. 2:**
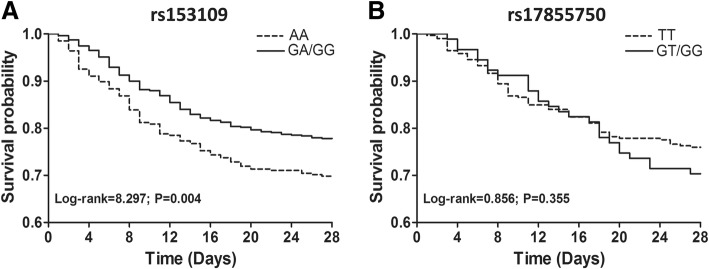
Kaplan-Meier survival analysis of patients with sepsis. The effect of *IL-27* rs153109 (**a**) and rs17855750 (**b**) on the 28-day survival of patients with sepsis was assessed using Kaplan-Meier survival analysis

The patients with sepsis were further stratified by 28-day mortality into two subgroups to evaluate the effect of *IL-27* SNPs on the clinical outcome of patients with sepsis. The frequencies of the AA genotypes and A allele at the *IL-27* SNP rs153109 A > G were significantly higher in 28-day non-surviving patients than that in the surviving patients (Table 5). Moreover, Kaplan-Meier survival analysis showed that 28-day survival in patients with sepsis carrying the rs153109 AA genotype was much worse than in the GA/GG genotype carriers (log-rank test 8.297, *P* = 0.004; Fig. 2A). Nevertheless, no significant differences were observed in the *IL-27* SNP at rs17855750 G > T (log-rank test 0.856, *P* = 0.335; Fig. 2B).

**Table 5:** Genotype and allele frequencies distribution in 28-day surviving and non-surviving patients with sepsis

*IL-27*	Survivors *n* (%)	Non-survivors *n* (%)	*P*	P*	OR (95% CI)
rs153109					
AA	235 (35.1)	99 (45.8)	0.019	0.025	–
GA	321 (48.0)	87 (40.3)	–	–	–
GG	113 (16.9)	30 (13.9)	–	–	–
AA/GA	556 (83.1)	186 (86.1)	0.297	0.297	0.794 (0.513–1.227)
GA/GG	434 (64.9)	117 (54.2)	0.005	0.020	1.563 (1.145–2.133)
A	791 (59.1)	285 (66.0)	–	–	1.000 (reference)
G	547 (40.9)	147 (34.0)	0.011	0.022	1.341 (1.068–1.682)
rs17855750					
TT	237 (78.7)	75 (73.5)	0.311	0.311	–
GT	59 (19.6)	23 (22.6)	–	–	–
GG	5 (1.7)	4 (3.9)	–	–	–
TT/GT	296 (98.3)	98 (96.1)	0.239	0.311	2.416 (0.636-9.180)
GT/GG	64 (21.3)	27 (26.5)	0.277	0.311	0.750 (0.446-1.261)
T	533 (88.5)	173 (84.8)	–	–	1.000 (reference)
G	69 (11.5)	31 (15.2)	0.162	0.311	0.722 (0.457-1.141)

*OR* odds ratio, *95% CI* 95% confidence interval

*False discovery rate-adjusted *P* value for multiple hypotheses testing using the Benjamini-Hochberg method

### The association between *IL-27* SNPs and *IL-27* expression in patients with sepsis and healthy controls

**Fig. 3:**
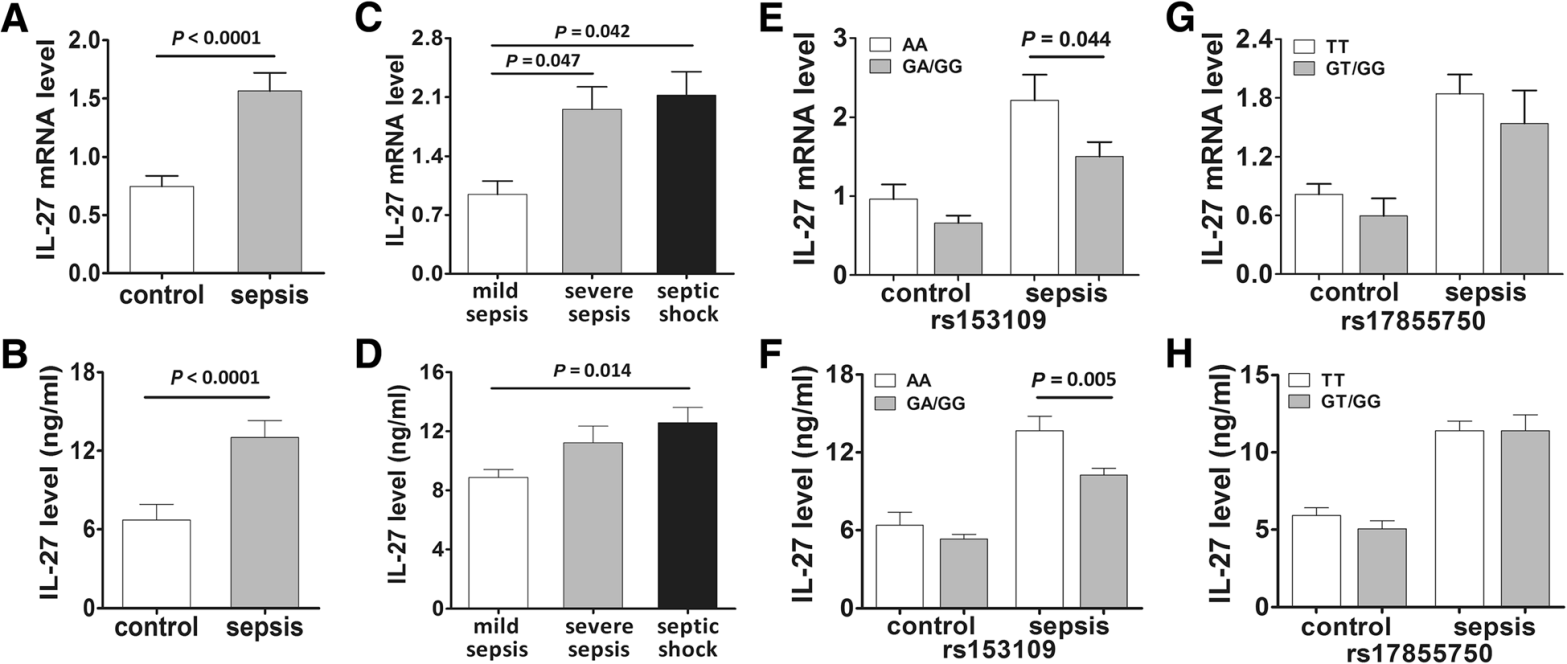
The expression of *IL-27* in patients with sepsis and healthy controls with different *IL-27* polymorphisms. The mRNA expression level and plasma concentration of IL-27 in patients with sepsis (*n* = 80) and healthy controls (*n* = 80) (**a**, **b**); the mRNA expression level and plasma concentration of IL-27 in mild sepsis, severe sepsis, and septic shock subgroups (**c**, **d**); the distribution of IL-27 expression levels in groups of patients with sepsis with different rs153109 genotypes (**e**, **f**) and different rs17855750 genotypes (**g**, **h**). The horizontal line represents the median expression level with each group. The error bar represents the standard error of the mean

A total of 80 patients with sepsis and 80 healthy individuals were randomly selected to evaluate the possible associations between the *IL-27* rs153109 or rs17855750 polymorphisms and *IL-27* expression in PBMCs. As presented in Fig. 3, significantly greater *IL-27* mRNA levels were found in patients with sepsis relative to the controls (Fig. 3A, *P* < 0.0001), and expression significantly was significantly greater with the development of sepsis (Fig. 3C, *P* = 0.047 for severe sepsis and *P* = 0.042 for septic shock). When the data were stratified by genotype, our results suggested that the *IL-27* mRNA levels in patients with sepsis carrying the AA genotype of rs153109 were significantly higher than those in patients with the GA/GG genotypes (Fig. 3E, *P* = 0.044). With regard to rs17855750, no significant difference in the *IL-27* mRNA expression was observed between different genotypes in patients with sepsis or healthy individuals (Fig. 3G, *P* > 0.05). Additionally, we detected the plasma concentration of IL-27 in these patients with sepsis and in controls using ELISA, which was consistent with the results from the gene expression analysis in PBMCs (Fig. 3).

### The effect of *IL-27* SNPs on *IL-27* expression in LPS-stimulated PBMCs and the promoter activity of the gene

**Fig. 4:**
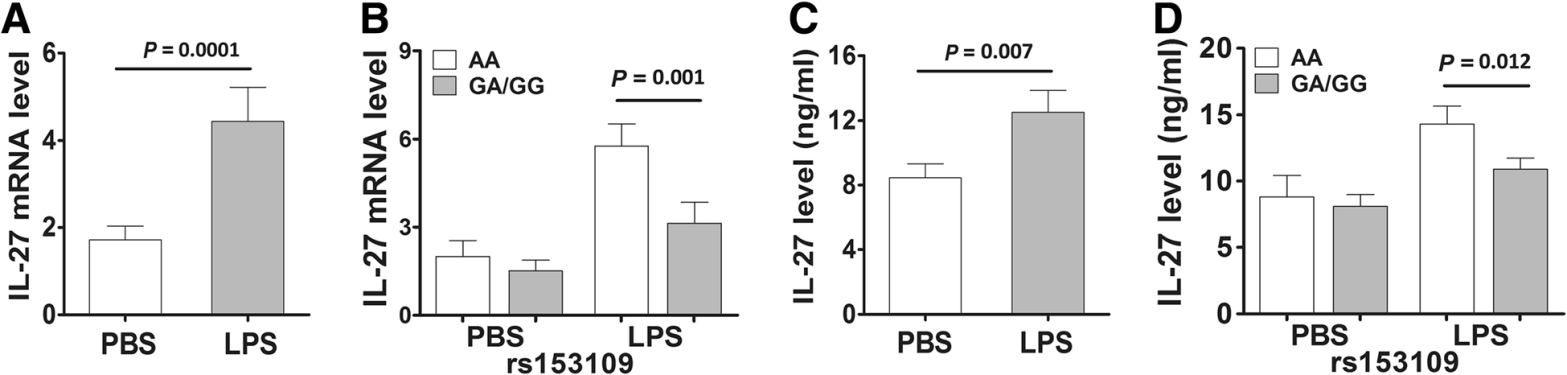
The *IL-27* expressions in the peripheral blood mononuclear cells (PBMCs) isolated from 55 healthy volunteers. The *IL-27* mRNA expression of PBMCs from 55 healthy individuals under lipopolysaccharide (LPS) stimulation (500 ng/mL) in groups with different rs153109 genotypes (**a**, **b**); the supernatant concentration of IL-27 under LPS stimulation in groups with different rs153109 genotypes (**c**, **d**). The horizontal line indicates the mean expression level in each genotype group. The error bar represents standard error of the mean

To validate the effect of the *IL-27* rs153109 and rs17855750 polymorphisms on *IL-27* expression at both the transcriptional and translational levels, we further measured the *IL-27* mRNA expression and protein production in PBMCs from 55 healthy subjects with different genotypes under LPS stimulation in vitro. As shown in Fig. 4, the *IL-27* mRNA expression and protein production were significantly greater in PBMCs carrying the rs153109 AA genotype compared to those with the GA/GG genotypes upon LPS stimulation (*P* = 0.001 and *P* = 0.012, respectively), but these differences were not observed without LPS induction (*P* > 0.05).

**Fig. 5:**
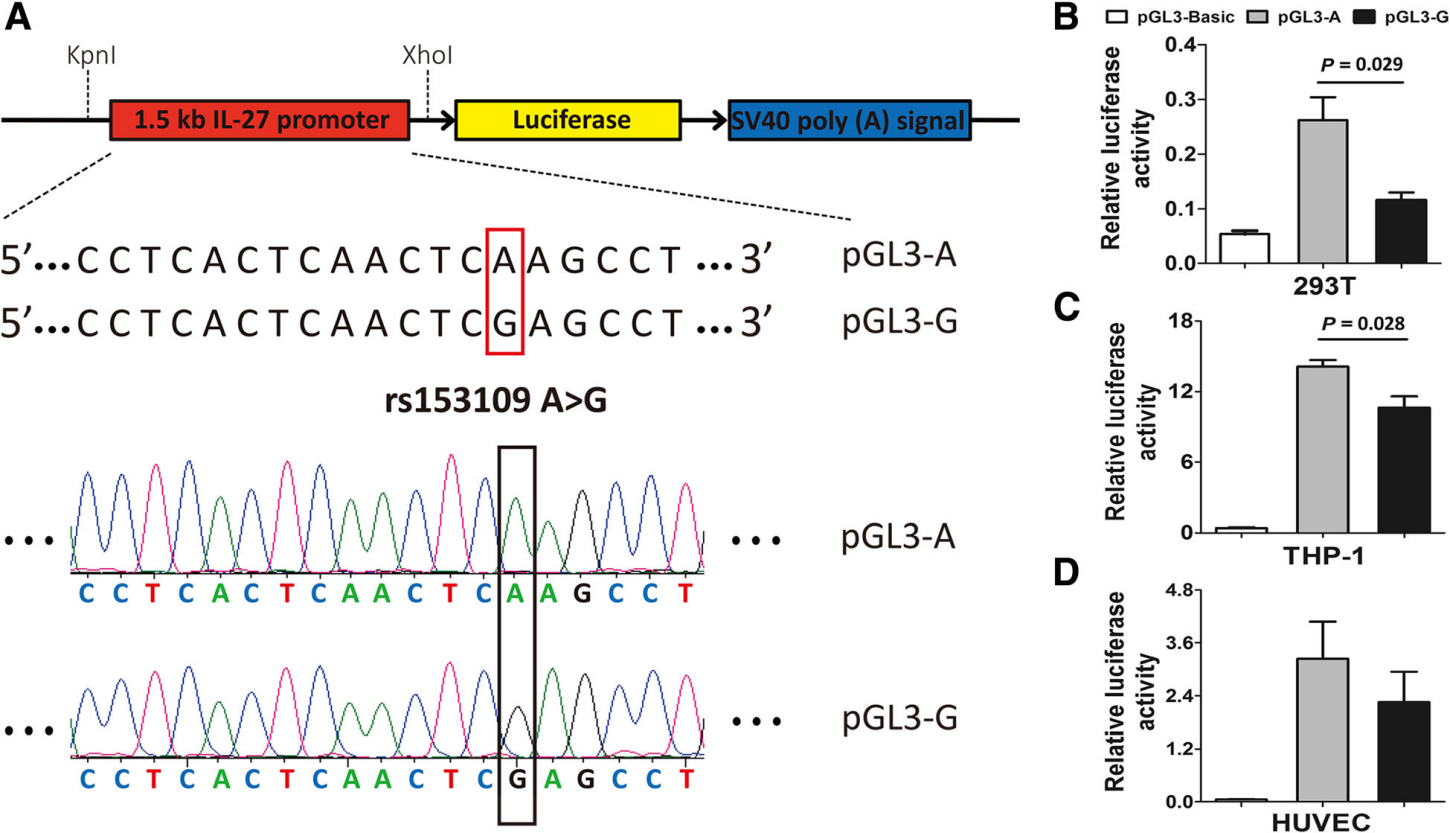
The *IL-27* plasmid constructs and functional promoter activities of rs153109 in 293 T, THP-1 and HUVECs. The promoter region of *IL-27* DNA sequence (2145 bp) carrying A or G of rs153109 A > G was cloned into pGL3 luciferase reporter vectors (**a**); the different *IL-27* plasmid constructs were transfected into 293 T, THP-1 and HUVEC cells for 48 h, and then the promoter activities were detected by dual-luciferase report assays (**b**, **c**, **d**). Three parallel samples were used in all transfections, and all experiments were performed in triplicate. The error bar represents standard error of the mean

Considering the location of the *IL-27* rs153109 polymorphism upstream of the transcription start site (− 964 bp), we further performed luciferase assays using different cell lines to evaluate the effect of the − 964 A-to-G variation on the promoter activities of the *IL-27* gene. As presented in Fig. 5, the pGL3 luciferase reporter vector containing the − 964 A allele exhibited significantly higher transcription activity of *IL-27* than the vector containing the − 964 G allele in 293 T and THP-1 cells (*P* = 0.029 and *P* = 0.028, respectively), but no significant differences were observed in HUVECs (*P* > .05).

### The effect of *IL-27* SNPs on the expression of related pro-inflammatory cytokines

**Fig. 6:**
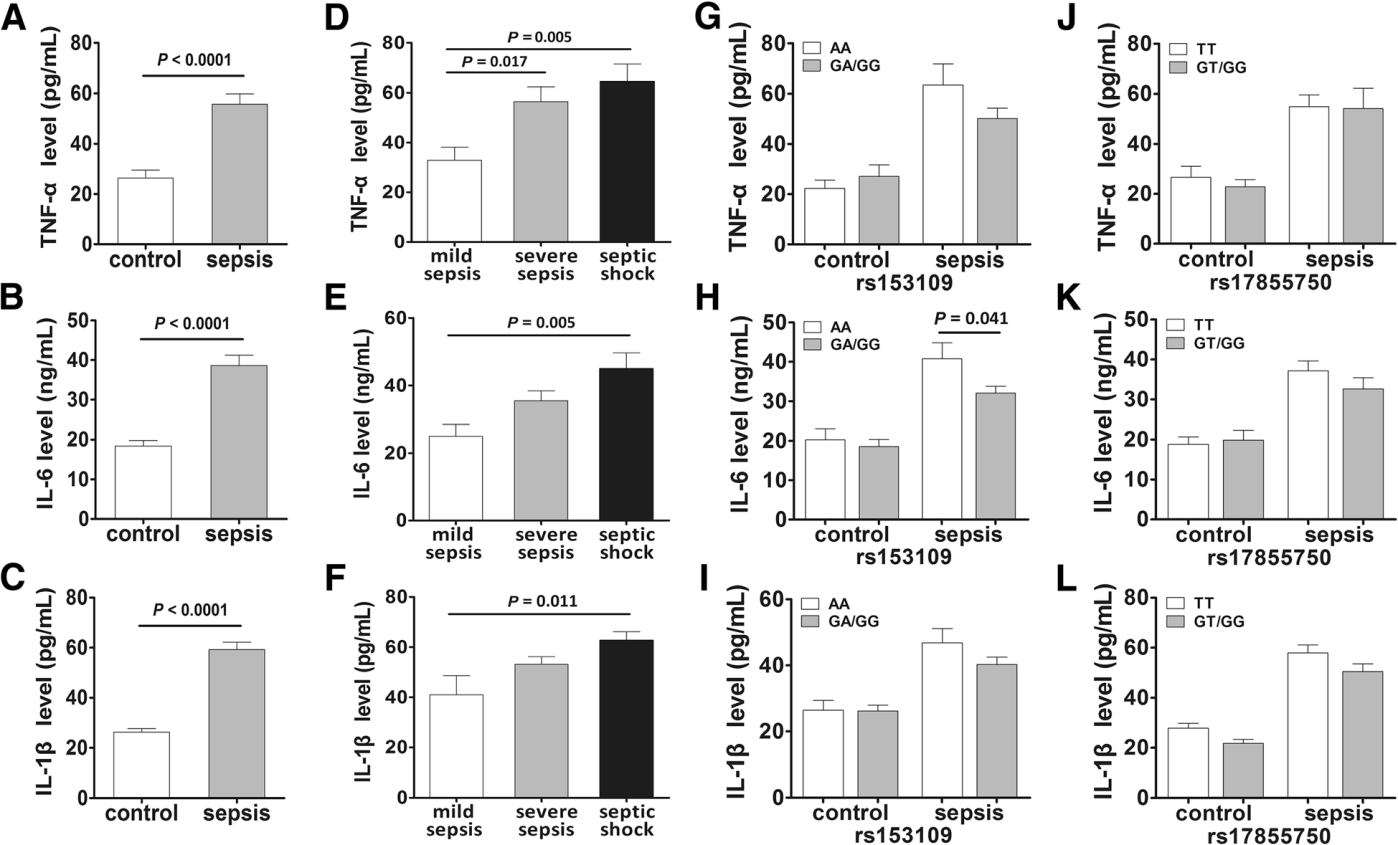
The plasma concentrations of several pro-inflammatory cytokines in patients with sepsis and healthy controls with different *IL-27* polymorphisms. The plasma concentrations of TNF-α, IL-6 and IL-1β in patients with sepsis (*n* = 80) and healthy controls (*n* = 80) (**a**, **b**, **c**); the plasma concentrations of TNF-α, IL-6 and IL-1β in mild sepsis, severe sepsis, and septic shock subgroups (**d**, **e**, **f**). The distribution of TNF-α, IL-6 and IL-1β levels in groups of patients with sepsis with different rs153109 genotypes (**g**, **h**, **i**) and different rs17855750 genotypes (**j**, **k**, **l**). The horizontal line indicates the median concentration within each group. The error bar represents standard error of the mean

We further measured the plasma concentrations of related pro-inflammatory cytokines in the selected 80 patients with sepsis and 80 healthy subjects to evaluate the effect of the *IL-27* SNPs on the production of these cytokines. As shown in Fig. 6, the TNF-α, IL-1β, and IL-6 concentrations in patients with sepsis were markedly greater compared to those in the controls, and the levels also increased with the development of sepsis. When stratified by the rs153109 and rs17855750 genotypes, only the IL-6 concentration was observed to be closely associated with the rs153109 polymorphism in patients with sepsis (*P* = 0.041).

**Fig. 7:**
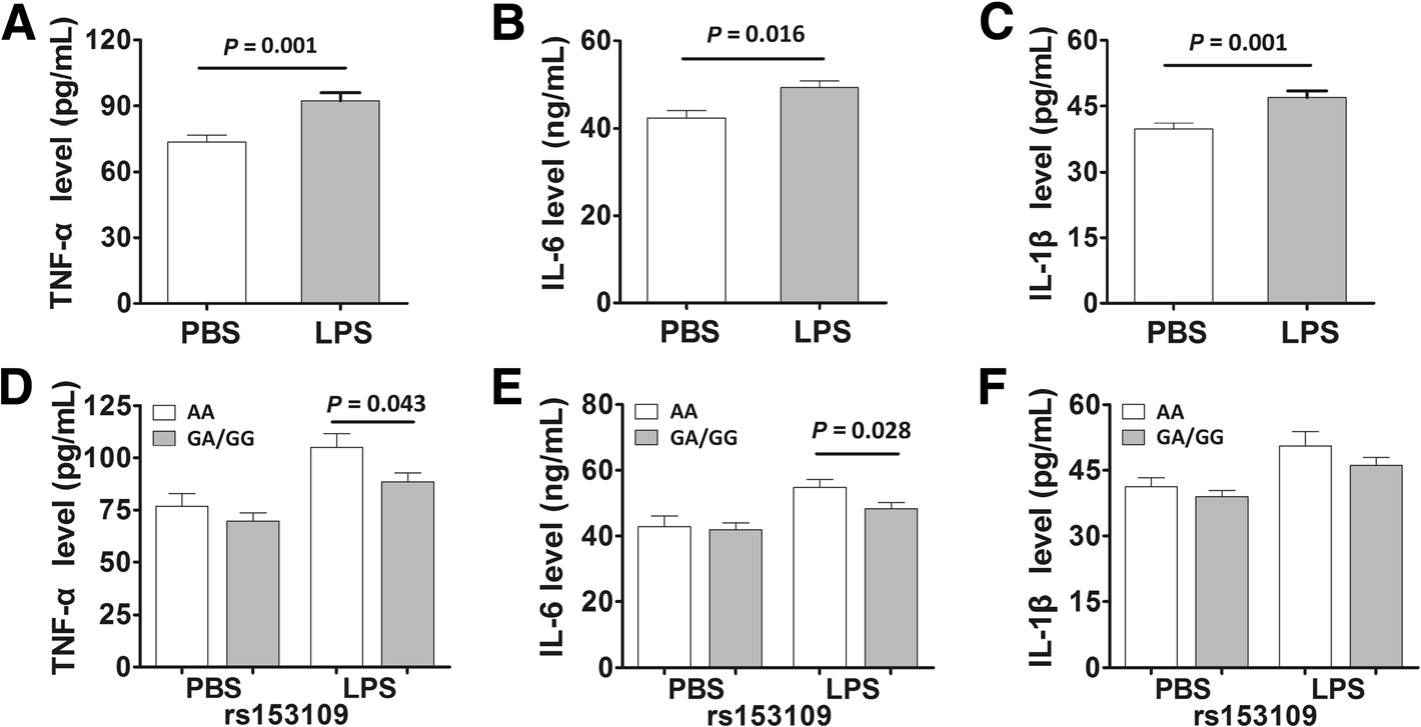
The *IL-27* rs153109 A > G polymorphism enhanced pro-inflammatory cytokine release of the human peripheral blood mononuclear cells (PBMCs). The PBMCs of 50 healthy volunteers were incubated in vitro and stimulated with 500 ng/mL lipolysaccharide (LPS) for 8 h. Then the supernatant concentration of TNF-α, IL-6 and IL-1β in groups with different rs153109 genotypes were measured (**a**-**f**). The horizontal line indicates the mean expression level within each genotype group. The error bar represents standard error of the mean

To confirm the effect of *IL-27* SNPs on these pro-inflammatory cytokines, we also measured the expression levels of these cytokines in the supernatants of PBMCs isolated from 55 healthy subjects with different genotypes under LPS stimulation in vitro. Our data suggested that the expression levels of TNF-α and IL-6 were significantly higher in the LPS-stimulated PBMCs with the rs153109 AA genotype than those with the GA/GG genotypes (Fig. 7, *P* = 0.043 and *P* = 0.028, respectively). However, no significant differences were found in IL-1β expression levels between PBMCs with different rs153109 genotypes (Fig. 7, *P* > 0.05).

### IL-27 enhances LPS-induced pro-inflammatory cytokine expression and cell apoptosis

**Fig. 8:**
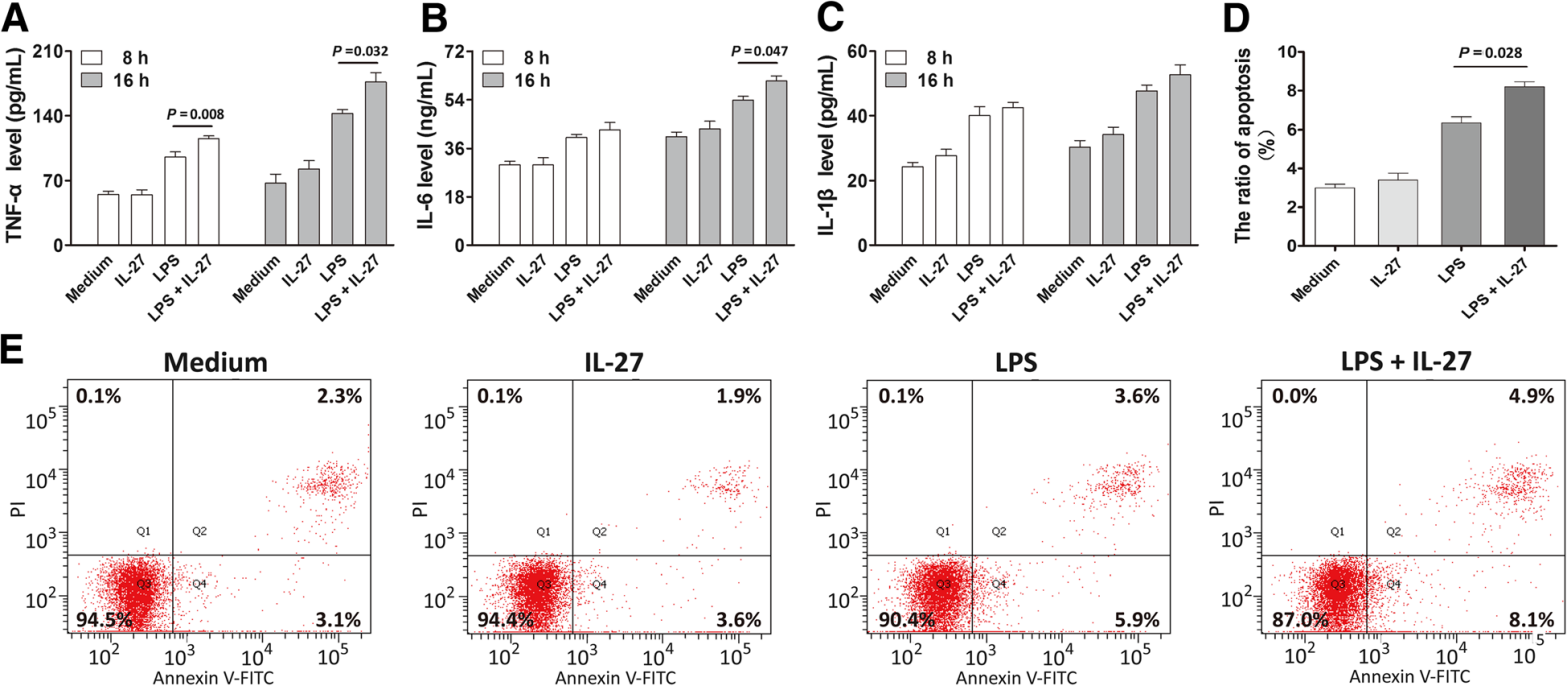
IL-27 enhances lipolysaccharide (LPS)-induced pro-inflammatory cytokine expression and cell apoptosis. The supernatant concentrations of TNF-α, IL-6, and IL-1β from THP-1 cells incubated with medium alone, IL-27 (100 ng/mL), LPS (500 ng/mL) or a combination of both for 8 and 16 h (**a**, **b**, **c**); Evaluation of apoptosis of THP-1 cells incubated with medium, IL-27 (100 ng/mL), LPS (500 ng/mL) or a combination of both for 16 h using annexin-V-fluorescein isothiocyanate (FITC)/propium iodide staining and flow cytometry (**d**, **e**). Bar graphs indicate the mean ± SEM for a minimum of three experiments, each performed in triplicate

We next evaluated the influence of IL-27 on the pro-inflammatory cytokine expression and cell apoptosis in THP-1 cells upon LPS stimulation. There was a significant increase in the LPS-stimulated expression of TNF-α and IL-6 following IL-27 treatment relative to the expression with IL-27 or LPS treatment alone (Fig. 8a, b). However, IL-27 treatment had no significant effect on the LPS-stimulated expression of IL-1β (Fig. 8c). In addition, the LPS-stimulated THP-1 cells displayed a significant increase in the apoptotic rate compared with the control cells (Fig. 8d, e). Most importantly, simultaneous treatment of cells with LPS and IL-27 resulted in a striking increase in the apoptotic rate relative to that of cells with IL-27 or LPS treatment alone.

## Discussion

Research so far has demonstrated that dysfunctional immune inflammatory responses are critical in promoting the development and progression of adverse outcomes in patients with sepsis [29–31]. The previous results from our group and evidence from other studies have indicated that SNPs in at least 38 genes from the pro-inflammatory signaling pathway are critical for determining individual differences in inflammatory responses and predisposition to sepsis susceptibility and progression [32–38]. In this study, for the first time, we investigated the association between sepsis and two functional polymorphisms (rs153109 and rs17855750) in the *IL-27* gene. No significant differences in the genotype/allele frequencies were found between patients with sepsis and healthy controls, which suggests that these *IL-27* SNPs may not be risk factors for the occurrence of sepsis. Further stratification showed that the rs153109 A allele was overrepresented in the patients with severe sepsis/septic shock relative to the patients with the sepsis subtype. Furthermore, significant differences in the rs153109 genotype/allele frequencies were observed between 28-day survivors and non-survivors among patients with sepsis, indicating that the rs153109 polymorphism may act as a risk factor for the severity of sepsis and poor outcome. The rs153109 (− 964 A > G) promoter polymorphism of *IL-27* may alter its gene transcription and cause alterations in the inflammatory responses, which ultimately result in the progression of sepsis from sepsis subtype to severe sepsis/septic shock and poor prognosis.

Accumulating evidence has demonstrated that the IL-12 family member IL-27 is pivotal in the pathogenic mechanisms of sepsis [11, 14–18]. IL-27, as an important regulatory cytokine mainly secreted by antigen-presenting cells upon exposure to microbial products and inflammatory stimuli, can regulate the duration and intensity of various T cell responses such as the expansion of Th1 cells, proliferation, and interferon (IFN)-c production in CD4+ T cells [39, 40]. Enhanced expression of *IL-27* has been identified in patients with sepsis and in a model of sepsis, which was closely correlated with disease severity and mortality [12, 13]. Our data suggest that both the transcriptional and translational levels of *IL-27* were overexpressed in patients with sepsis, which corroborates several previous studies. Wong et al*.* identified *IL-27* as a candidate diagnostic gene for sepsis, by genome-wide expression analysis [41, 42]. Other studies have indicated that elevated IL-27 strongly correlates with early-onset sepsis and might provide additional diagnostic value for sepsis, alone or in combination with PCT [43, 44]. In our study IL-27 exerted a significant effect on the progression of sepsis and might also serve as an indicator of disease severity as it increased with the development of sepsis.

Recent genetic association studies have confirmed that the *IL-27* rs153109 polymorphism affects mRNA expression and influences patient predisposition to various inflammation-related diseases [21–23, 45, 46]. A recent study showed that the rs153109 promoter polymorphism of *IL-27* probably influences gene transcription by altering its binding to transcription factors and that the AA genotype or A allele confers susceptibility to asthma [47]. Shen et al. discovered that the TT genotype and T allele of rs178855750 polymorphisms located in exon 2 of the *IL-27* gene are closely associated with allergic rhinitis [24]. Other studies have indicated that the A allele of the *IL-27* rs153109 polymorphism is associated with increased risk of Crohn’s disease and ulcerative colitis in a Korean population, while it is associated with resistance to Crohn’s disease in a Chinese Han population [48, 49]. Nevertheless, the role of these *IL-27* polymorphisms has not been determined in the pathogenesis of sepsis.

In this study, our data showed that the AA risk genotype or A allele at *IL-27* rs153109 was overrepresented in the subgroups of severe sepsis/septic shock compared to the subgroup of sepsis subtype, indicating that rs153109 A > G is involved in promoting the progression of sepsis from sepsis subtype to severe sepsis/septic shock. The frequencies of rs153109 AA genotype similarly increased in 28-day non-surviving patients with sepsis compared to 28-day survivors, and the 28-day survival in patients with sepsis with the AA genotype exhibited was worse than those carrying the GA/GG genotypes, which further supports the possibility that the rs153109 A > G is a risk factor for the progression of sepsis and poor prognosis. Furthermore, IL-27 expression in patients with sepsis carrying the rs153109 AA genotype significantly increased compared to that of the GA/GG genotype carriers, suggesting that this SNP might be involved in the pathogenesis of sepsis by altering its gene transcription. However, another *IL-27* polymorphism at rs17855750 G > T did not significantly affect sepsis susceptibility or progression.

The importance of functional evaluation of a genetic variant in the context of a disease has been emphasized in recent years [50, 51]. Mechanistic analysis of histone modifications within the promoter region of *IL-27* revealed the presence of H3K4me3 [52] and H3K27ac [53]. The rs153109 A > G polymorphism located 964 bp upstream of the transcription start site of the *IL-27* gene might increase the H3K4me3 and H3K27ac modifications, thereby promoting the transcriptional activity of the *IL-27* gene [21]. We performed luciferase assays using different types of cells to evaluate the effect of the − 964 A-to-G variation on the promoter activities of the *IL-27* gene and confirmed that the sepsis-associated A risk allele of rs153109 significantly increased the promoter activity in 293 T and THP-1 cells. To further validate this result, we evaluated the effect of rs153109 on *IL-27* expression in PBMCs upon LPS stimulation in vitro. Our data showed that the LPS-stimulated PBMCs with the rs153109 AA genotype exhibited significantly increased expression of *IL-27* compared to those with the GA/GG genotypes, as predicted. Nevertheless, no significant difference was found in *IL-27* expression between the AA and GA/GG genotypes of rs153109 without LPS induction. Our results infer that the genetic effect of the − 964 A-to-G variation might make a real difference following inflammatory stimuli or stress, which also suggests that the *IL-27* rs153109 SNP influences the sepsis severity rather than the onset of sepsis.

IL-27 mediates its biological function through ligation to the IL-27RA/gp130 receptor and plays pivotal roles in various T cell subsets, activating signaling through the mitogen-activated protein kinase (MAPK) and Janus kinase (JAK)/STAT pathways, following NF-κB activation [54]. This molecule also induces native T cells to differentiate into the Th1 subset via intercellular adhesion molecule-1/lymphocyte function-associated antigen 1 (LFA-1)/extracellular signal-related kinase (ERK)1/2, and p38 MAPK/T-bet signaling [55]. Recently, increasing evidence has identified IL-27 as a strong inducer of chemokines and pro-inflammatory cytokines, including TNF-α, IL-6, IL-1β, macrophage inflammatory protein (MIP)-1β, and MIP-1α by activated neutrophils, monocytes, and macrophages [56]. Gregersen et al. indicated that IL-27 increased NLRP3 inflammasome activation in monocytes with enhanced release of IL-1β [57]. Other studies have demonstrated that IL-27 enhances the production of these pro-inflammatory cytokines via activation of toll-like receptor (TLR)4 signaling [16, 58]. Consistently, our data showed that IL-27 treatment significantly enhanced TNF-α and IL-6 secretion and apoptosis of THP-1 cells upon LPS stimulation, which further demonstrated that IL-27 is critical in the pro-inflammatory responses during sepsis. Then, we detected the expression levels of IL-1β, IL-6 and TNF-α in patients with sepsis and healthy subjects to evaluate the influence of the functional *IL-27* SNP on the production of these related cytokines. However, only the IL-6 level was closely associated with the rs153109 polymorphism in patients with sepsis. Notably, the in vitro LPS-stimulated experiment showed that the sepsis-associated AA risk genotype significantly increased TNF-α and IL-6 secretion in PBMCs upon exposure to LPS in vitro. These results further supported the hypothesis that the sepsis-associated A risk allele of the rs153109 functional SNP enhanced promoter activity and gene transcription of *IL-27*, thereby directly or indirectly promoting sepsis-induced inflammatory responses, which ultimately resulted in the development of sepsis from sepsis subtype to severe sepsis/septic shock and poor prognosis, with potentially important therapeutic implications. The exact mechanism needs to be studied further.

Several limitations of this study should be acknowledged. First, we could not eliminate any potential selection bias in the enrolled population due to the wide inclusion criteria and regional central referring hospital. Second, the sample size in this study was insufficient, and all subjects were of Chinese Han nationality. Therefore, further biological studies with larger populations and different ethnic backgrounds are required to validate our tentative conclusions. Third, although we excluded patients with sepsis with specific diseases to attain greater homogeneity in the samples, it remains possible that the pre-existing conditions of the patients with sepsis may make a difference in the genetic results and observations in this case-control study. In addition, other functional polymorphisms may interfere with *IL-27* expression, and these integrated effects should be studied for better estimation of individual risk of the onset or development of sepsis.

## Conclusions

In this study, for the first time, we provided evidence indicating a significant association between the *IL-27* polymorphism and the progression of sepsis in a Chinese Han population. The sepsis-associated rs153109 (− 964 A > G) SNP within the promoter region of *IL-27* was capable of increasing the transcription of the *IL-27* gene by regulating its promoter activities, upregulating both transcription and translation of *IL-27* and then enhancing the secretion of pro-inflammatory cytokines, which were closely associated with the severity of sepsis and poor outcome. These results might to some extent explain the heterogeneity of the clinical course in patients with sepsis.

## Additional files

Additional file 1: CONSORT flowchart of the study. (PDF 210 kb)

Additional file 2: The prevalent comorbidities of patients with sepsis. (DOCX 13 kb)

Additional file 3: The Hardy-Weinberg equilibrium of the two IL-27 polymorphisms. (DOCX 12 kb)

## Abbreviations

APACHE II: Acute Physiology and Chronic Health Evaluation II
bp: Base pairs
cDNA: Complementary deoxyribonucleic acid
COPD: Chronic obstructive pulmonary disease
ELISA: Enzyme-linked immunosorbent assay
FBS: Fetal bovine serum
HUVEC: Human umbilical vein endothelial cell
ICU: Intensive care unit
IL-1β: Interleukin-1 beta
IL-27: Interleukin-27
IL-6: Interleukin-6
LPS: Lipopolysaccharide
mRNA: Messenger RNA
PBMC: Peripheral blood mononuclear cell
PBS: Phosphate-buffered saline
QUANTO: Quantity adjusting option
RT-PCR: Real-time polymerase chain reaction
siRNA: Small interfering ribonucleic acid
SNP: Single nucleotide polymorphism
THP-1: Human acute monocytic leukemia cell line
TNF-a: Tumor necrosis factor alpha
UTR: Untranslated region

## References

[1] Singer M, Deutschman CS, Seymour CW (2016). The Third International Consensus definitions for sepsis and septic shock (Sepsis-3). JAMA.

[2] Hotchkiss RS, Sherwood ER (2015). Immunology. Getting sepsis therapy right. Science.

[3] Stevenson EK, Rubenstein AR, Radin GT (2014). Two decades of mortality trends among patients with severe sepsis: a comparative meta-analysis. Crit Care Med.

[4] Zhang M, Zhao Y, Liu Q (2017). Tumor necrosis factor-alpha -308G/A and -238G/A polymorphisms are associated with increased risks of sepsis: evidence from an updated meta-analysis. APMIS.

[5] Jimenez-Sousa MA, Medrano LM, Liu P (2017). IL-6 rs1800795 polymorphism is associated with septic shock-related death in patients who underwent major surgery: a preliminary retrospective study. Ann Intensive Care.

[6] Montoya-Ruiz C, Jaimes FA, Rugeles MT (2016). Variants in LTA, TNF, IL1B and IL10 genes associated with the clinical course of sepsis. Immunol Res.

[7] Hunter CA, Kastelein R (2012). Interleukin-27: balancing protective and pathological immunity. Immunity.

[8] Aparicio-Siegmund S, Garbers C (2015). The biology of interleukin-27 reveals unique pro- and anti-inflammatory functions in immunity. Cytokine Growth Factor Rev.

[9] Duan Y, Jia Y, Wang T (2015). Potent therapeutic target of inflammation, virus and tumor: focus on interleukin-27. Int Immunopharmacol.

[10] Wynick C, Petes C, Gee K (2014). Interleukin-27 mediates inflammation during chronic disease. J Interf Cytokine Res.

[11] Neurath MF (2007). New therapies for sepsis: focus on the interleukin (IL)12 family member IL27. Ann Rheum Dis.

[12] Rinchai D, Khaenam P, Kewcharoenwong C (2012). Production of interleukin-27 by human neutrophils regulates their function during bacterial infection. Eur J Immunol.

[13] Roewe J, Higer M, Riehl DR (2017). Neuroendocrine modulation of IL-27 in macrophages. J Immunol.

[14] Cao J, Xu F, Lin S (2014). IL-27 controls sepsis-induced impairment of lung antibacterial host defence. Thorax.

[15] Bosmann M, Strobl B, Kichler N (2014). Tyrosine kinase 2 promotes sepsis-associated lethality by facilitating production of interleukin-27. J Leukoc Biol.

[16] Guzzo C, Ayer A, Basta S (2012). IL-27 enhances LPS-induced proinflammatory cytokine production via upregulation of TLR4 expression and signaling in human monocytes. J Immunol.

[17] Bosmann M, Russkamp NF, Strobl B (2014). Interruption of macrophage-derived IL-27(p28) production by IL-10 during sepsis requires STAT3 but not SOCS3. J Immunol.

[18] Wirtz S, Tubbe I, Galle PR (2006). Protection from lethal septic peritonitis by neutralizing the biological function of interleukin 27. J Exp Med.

[19] Oh JY, Sim JK, Jung WJ (2015). Association between interleukin-27 polymorphisms and pulmonary tuberculosis. Int J Tuberc Lung Dis.

[20] Ghavami Alireza, Fathpour Gholamreza, Amirghofran Zahra (2017). Association of IL-27 rs153109 and rs17855750 Polymorphisms with Risk and Response to Therapy in Acute Lymphoblastic Leukemia. Pathology & Oncology Research.

[21] Yu W, Zhang K, Wang Z (2017). Functional variant in the promoter region of IL-27 alters gene transcription and confers a risk for ulcerative colitis in northern Chinese Han. Hum Immunol.

[22] Paradowska-Gorycka A, Raszkiewicz B, Jurkowska M (2014). Association of single nucleotide polymorphisms in the IL27 gene with rheumatoid arthritis. Scand J Immunol.

[23] Huang N, Liu L, Wang XZ (2008). Association of interleukin (IL)-12 and IL-27 gene polymorphisms with chronic obstructive pulmonary disease in a Chinese population. DNA Cell Biol.

[24] Shen Y, Yuan XD, Hu D (2014). Association between interleukin-27 gene polymorphisms and susceptibility to allergic rhinitis. Hum Immunol.

[25] Shi J, Yuan M, Liu S (2016). Correlation of IL-27 genetic polymorphisms with the risk and survival of cervical cancer in a Chinese Han population. Tumour Biol.

[26] Levy MM, Fink MP, Marshall JC (2003). 2001 SCCM/ESICM/ACCP/ATS/SIS International Sepsis Definitions Conference. Intensive Care Med.

[27] Dellinger RP, Levy MM, Rhodes A (2013). Surviving Sepsis Campaign: international guidelines for management of severe sepsis and septic shock, 2012. Intensive Care Med.

[28] Cui L, Gao Y, Xie Y (2015). An ADAM10 promoter polymorphism is a functional variant in severe sepsis patients and confers susceptibility to the development of sepsis. Crit Care.

[29] Wiersinga Willem Joost, Leopold Stije J, Cranendonk Duncan R, van der Poll Tom (2013). Host innate immune responses to sepsis. Virulence.

[30] Steeland S, Van Ryckeghem S, Vandewalle J (2018). Simultaneous inhibition of tumor necrosis factor receptor 1 and matrix metalloproteinase 8 completely protects against acute inflammation and sepsis. Crit Care Med.

[31] Lewis DH, Chan DL, Pinheiro D (2012). The immunopathology of sepsis: pathogen recognition, systemic inflammation, the compensatory anti-inflammatory response, and regulatory T cells. J Vet Intern Med.

[32] Shao Y, Chen F, Chen Y (2017). Association between genetic polymorphisms in the autophagy-related 5 gene promoter and the risk of sepsis. Sci Rep.

[33] Shao Y, He J, Chen F (2016). Association study between promoter polymorphisms of ADAM17 and progression of sepsis. Cell Physiol Biochem.

[34] He J, Chen Y, Lin Y (2017). Association study of MCP-1 promoter polymorphisms with the susceptibility and progression of sepsis. PLoS One.

[35] Shao Y, Shao X, He J (2017). The promoter polymorphisms of receptor for advanced glycation end products were associated with the susceptibility and progression of sepsis. Clin Genet.

[36] Gupta DL, Nagar PK, Kamal VK (2015). Clinical relevance of single nucleotide polymorphisms within the 13 cytokine genes in North Indian trauma hemorrhagic shock patients. Scand J Trauma Resusc Emerg Med.

[37] Zhang H, Lu Y, Sun G (2017). The common promoter polymorphism rs11666254 downregulates FPR2/ALX expression and increases risk of sepsis in patients with severe trauma. Crit Care.

[38] David VL, Ercisli MF, Rogobete AF (2017). Early prediction of sepsis incidence in critically ill patients using specific genetic polymorphisms. Biochem Genet.

[39] Bosmann M, Ward PA (2013). Modulation of inflammation by interleukin-27. J Leukoc Biol.

[40] Qiu HN, Liu B, Liu W (2016). Interleukin-27 enhances TNF-alpha-mediated activation of human coronary artery endothelial cells. Mol Cell Biochem.

[41] Hanna WJ, Berrens Z, Langner T (2015). Interleukin-27: a novel biomarker in predicting bacterial infection among the critically ill. Crit Care.

[42] Wong HR, Cvijanovich NZ, Hall M (2012). Interleukin-27 is a novel candidate diagnostic biomarker for bacterial infection in critically ill children. Crit Care.

[43] He Y, Du WX, Jiang HY (2017). Multiplex cytokine profiling identifies interleukin-27 as a novel biomarker for neonatal early onset sepsis. Shock.

[44] Wong HR, Lindsell CJ, Lahni P (2013). Interleukin 27 as a sepsis diagnostic biomarker in critically ill adults. Shock.

[45] Dehghanzadeh R, Babaloo Z, Sakhinia E (2016). IL-27 Gene polymorphisms in Iranian patients with Behcet's disease. Clin Lab.

[46] Zhang D, Ma M, Yang Y (2016). Association between polymorphisms in IL27 and risk for CHD in a Chinese population. Cardiol Young.

[47] Chae SC, Li CS, Kim KM (2007). Identification of polymorphisms in human interleukin-27 and their association with asthma in a Korean population. J Hum Genet.

[48] Li CS, Zhang Q, Lee KJ (2009). Interleukin-27 polymorphisms are associated with inflammatory bowel diseases in a Korean population. J Gastroenterol Hepatol.

[49] Wang Z, Wang L, Fan R (2014). Association of IL-27 gene three polymorphisms with Crohn's disease susceptibility in a Chinese Han population. Int J Clin Exp Pathol.

[50] Wang S, Wen F, Wiley GB (2013). An enhancer element harboring variants associated with systemic lupus erythematosus engages the TNFAIP3 promoter to influence A20 expression. PLoS Genet.

[51] Wang S, Wiley GB, Kelly JA (2015). Disease mechanisms in rheumatology--tools and pathways: defining functional genetic variants in autoimmune diseases. Arthritis Rheumatol.

[52] Okitsu CY, Hsieh JC, Hsieh CL (2010). Transcriptional activity affects the H3K4me3 level and distribution in the coding region. Mol Cell Biol.

[53] Creyghton MP, Cheng AW, Welstead GG (2010). Histone H3K27ac separates active from poised enhancers and predicts developmental state. Proc Natl Acad Sci U S A.

[54] Wirtz S, Becker C, Fantini MC (2005). EBV-induced gene 3 transcription is induced by TLR signaling in primary dendritic cells via NF-kappa B activation. J Immunol.

[55] Owaki T, Asakawa M, Fukai F (2006). IL-27 induces Th1 differentiation via p38 MAPK/T-bet- and intercellular adhesion molecule-1/LFA-1/ERK1/2-dependent pathways. J Immunol.

[56] Guzzo C, Che Mat NF, Gee K (2010). Interleukin-27 induces a STAT1/3- and NF-kappaB-dependent proinflammatory cytokine profile in human monocytes. J Biol Chem.

[57] Gregersen I, Sandanger O, Askevold ET (2017). Interleukin 27 is increased in carotid atherosclerosis and promotes NLRP3 inflammasome activation. PLoS One.

[58] Petes C, Wynick C, Guzzo C (2017). IL-27 enhances LPS-induced IL-1beta in human monocytes and murine macrophages. J Leukoc Biol.

