# Supplementary material for: The interleukin-27 -964A>G polymorphism enhances sepsis-induced inflammatory responses and confers susceptibility to the development of sepsis
Source: Crit Care. 2018 Sep 30;22:248. doi: 10.1186/s13054-018-2180-0 (PMC6164187; doi:10.1186/s13054-018-2180-0)
Supplement: Supplementary file 1 — CONSORT flowchart of the study. (PDF 210 kb) [file 13054_2018_2180_MOESM1_ESM.pdf]

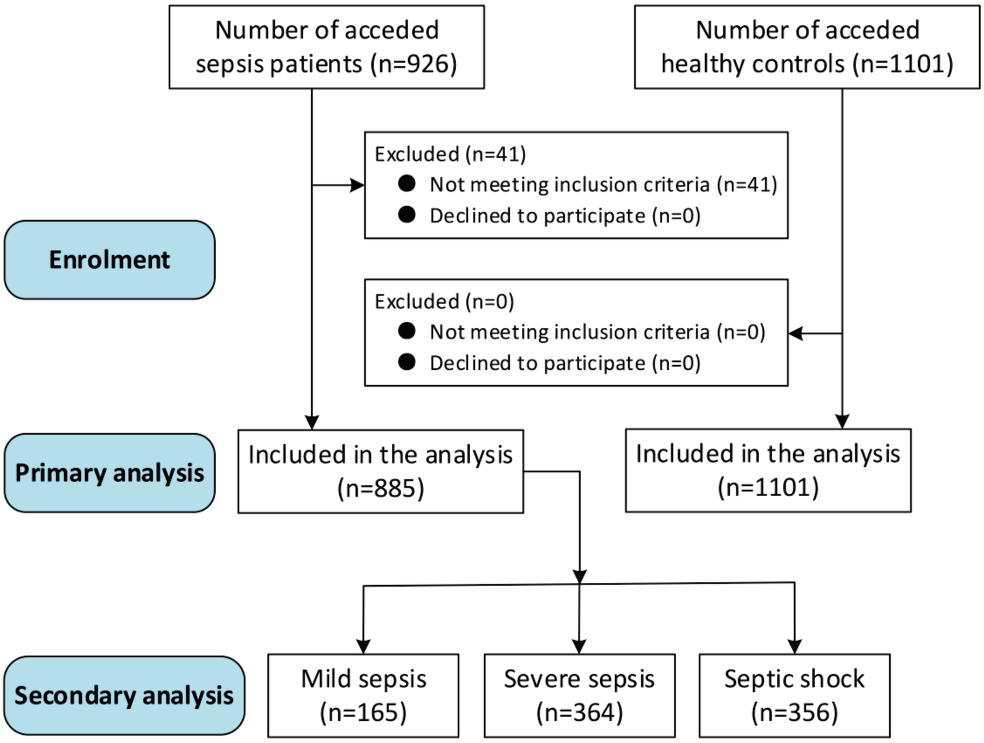

Additional file 1: CONSORT flowchart of the study. The figure shows the CONSORT flowchart of the study.
