# Supplementary material for: The interleukin-27 -964A>G polymorphism enhances sepsis-induced inflammatory responses and confers susceptibility to the development of sepsis
Source: Crit Care. 2018 Sep 30;22:248. doi: 10.1186/s13054-018-2180-0 (PMC6164187; doi:10.1186/s13054-018-2180-0)
Supplement: Supplementary file 2 — The prevalent comorbidities of patients with sepsis. (DOCX 13 kb) [file 13054_2018_2180_MOESM2_ESM.docx]

**Additional file 2:** The prevalent comorbidities of patients with sepsis.

| **Comorbidities** | **N (%)** |
| --- | --- |
| Severe pneumonia | 198 (22.4) |
| Hypertension | 175 (19.8) |
| Cerebrovascular disease | 153 (17.3) |
| Trauma | 121 (13.7) |
| Renal disease | 91 (10.3) |
| COPD | 89 (10.1) |
| Ischemic cardiopathy | 70 (7.9) |
| Chronic liver disease | 58 (6.6) |
| Biliary tract disease | 57 (6.4) |
| Gaslrointestinal perforation | 49 (5.5) |
| Severe acute pancreatitis | 26 (2.9) |
