# Supplementary material for: The interleukin-27 -964A>G polymorphism enhances sepsis-induced inflammatory responses and confers susceptibility to the development of sepsis
Source: Crit Care. 2018 Sep 30;22:248. doi: 10.1186/s13054-018-2180-0 (PMC6164187; doi:10.1186/s13054-018-2180-0)
Supplement: Supplementary file 3 — The Hardy-Weinberg equilibrium of the two IL-27 polymorphisms. (DOCX 12 kb) [file 13054_2018_2180_MOESM3_ESM.docx]

**Additional file 3:** The Hardy-Weinberg equilibrium of the two IL-27 polymorphisms

| **Hardy-Weinberg P** | **Sepsis** | **Control** |
| --- | --- | --- |
| rs153109 | 0.327 | 0.075 |
| rs17855750 | 0.200 | 0.683 |
